# Coupling of a specific photoreactive triple-helical peptide to crosslinked collagen films restores binding and activation of DDR2 and VWF

**DOI:** 10.1016/j.biomaterials.2018.07.050

**Published:** 2018-11

**Authors:** Jean-Daniel Malcor, Victoria Juskaite, Despoina Gavriilidou, Emma J. Hunter, Natalia Davidenko, Samir Hamaia, Sanjay Sinha, Ruth E. Cameron, Serena M. Best, Birgit Leitinger, Richard W. Farndale

**Affiliations:** a Department of Biochemistry, University of Cambridge, Cambridge, CB2 1QW, UK; b National Heart and Lung Institute, Imperial College London, London, UK; c Department of Materials Science and Metallurgy, University of Cambridge, Cambridge, UK; d Division of Medicine and Wellcome Trust – Medical Research Council Cambridge Stem Cell Institute, University of Cambridge, Cambridge, UK

**Keywords:** Collagen, Triple-helical peptide, Biomimetic material, Discoidin domain receptor 2, von Willebrand factor, Biomaterial functionalization

## Abstract

Collagen-based scaffolds may require chemical crosslinking to achieve mechanical properties suitable for tissue engineering. Carbodiimide treatment, often used for this purpose, consumes amino acid side chains required for receptor recognition, thus reducing cell–collagen interaction. Here, we restore recognition and function of both von Willebrand Factor (VWF) and Discoidin Domain Receptor 2 (DDR2) to crosslinked collagen films by derivatisation with a specific triple-helical peptide (THP), an approach previously applied to integrin-mediated cellular adhesion. The THP contained the collagen III-derived active sequence, GPRGQOGVNleGFO, conjugated to a photoreactive moiety, diazirine, allowing UV-dependent covalent coupling to collagen films. Crosslinking of collagen films attenuated the binding of recombinant VWF A3 domain and of DDR2 (as the GST and Fc fusions, respectively), and coupling of the specific THP restored their attachment. These derivatised films supported activation of DDR2 expressed in either COS-7 or HEK293 cells, reflected by phosphorylation of tyrosine 740, and VWF-mediated platelet deposition from flowing blood was restored. Further, such films were able to increase low-density lipoprotein uptake in vascular endothelial cells, a marker for endothelial phenotype. Thus, covalent linkage of specific THPs to crosslinked collagen films i) restores their cognate protein binding, ii) triggers the corresponding cellular responses, and iii) demonstrates the broad applicability of the approach to a range of receptors for applications in regenerative medicine.

## Graphical abstract

**Figure:**
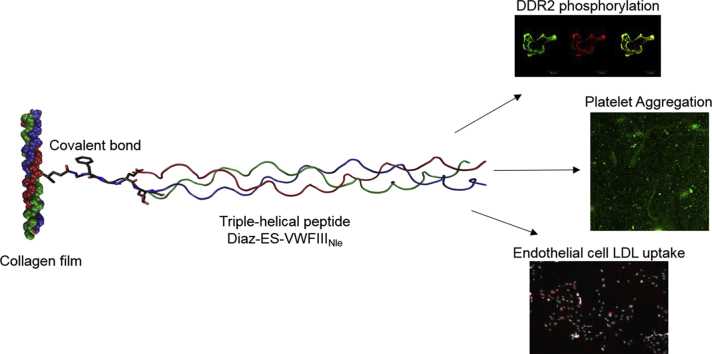

## 1 Introduction

Collagen is widely used in tissue engineering [[1], [2], [3]]. It is the most abundant protein in the human body and is a major constituent of the extracellular matrix (ECM), providing natural structural and biological support for cells [4]. The mechanical properties of collagen-based biomaterials can be tailored to match the strength, stiffness and architecture of the host tissue. Chemical crosslinking of a collagen-based scaffold using 1-ethyl-3-(3-dimethylaminopropyl)carbodiimide (EDC) and *N*-hydroxysuccinimide (NHS), is widely used to increase its stiffness and to resist swelling in biological media [[5], [6], [7]]. However, we have previously shown that such crosslinking ablated the recognition of the collagen-binding integrins (α_1_β_1_, α_2_β_1_, α_10_β_1_ and α_11_β_1_) and led to a dramatic loss of cell adhesion compared to native type I collagen [[8], [9], [10], [11], [12]], as a result of consumption of the glutamate residues in integrin binding Gxx'GEx'' motifs [13,14] in forming isopeptide bonds with lysines. Other collagen receptors might also be affected if their binding sequences contain aspartate, glutamate or lysine residues, or if they are adjacent to such EDC/NHS-sensitive residues. We show in this study that DDR2 and the A3 domain of VWF are affected by EDC/NHS crosslinking due to the presence of adjacent glutamate and lysine, even though their immediate binding site in collagen lacks such residues. For use in regenerative medicine, these materials must support cell attachment, subsequent differentiation and proliferation to fulfil their physiological function in the target tissue. Previously, we reported a methodology to derivatise collagen films with photoreactive triple-helical peptides (THPs) containing the GFOGER integrin binding motif [15]. THPs mimic the natural structure of collagen by adopting a triple helix conformation, essential for recognition by collagen binding receptors. THPs, however, remain rarely used in tissue engineering and, although examples can be found on various surfaces [16,17], their incorporation into collagen-based materials has only been investigated in this laboratory, to our knowledge. In our hands, this led to complete restoration of integrin mediated cell binding and spreading to EDC/NHS crosslinked collagen films [15].

We aim to apply the same technology to target other collagen-binding proteins using motifs from collagens that are specific for other receptors or matrix components. Previously, we synthesized THP libraries, called Toolkits, composed of an active guest sequence flanked by five GPP host triplets that form a triple helix [18]. Screening of collagen binding proteins against Toolkits II and III (encompassing the sequences of collagen II and III respectively), led to the identification of a THP ligand for both Discoidin Domain Receptors (DDRs) [19,20] and von Willebrand Factor (VWF) [18,[21], [22], [23]]. The shared binding motif GPRGQOGVMGFO, referred to as VWFIII in the literature, is derived from the active sequence of Toolkit III-23, GPOGPSGPRGQOGVMGFOGPKGNDGAO. A similar motif, G**A**RGQOGVMGFO, is present in collagen II, and an equivalent sequence, G**A**RGQ**A**GVMGFO, occurs in the α1 chain of collagen I (bold text represents differences in sequence).

Discoidin Domain Receptors, like the collagen-binding integrins, constitute a major collagen-binding receptor family; DDR1 and DDR2 are widely distributed in mammalian tissues and play important roles in development and wound repair [20,24,25]. They are tyrosine kinase receptors and their auto-phosphorylation is induced by binding to various types of collagen, including collagens I, II and III [19,22,26,27]. Finally, DDR2 is suspected of being present in cardiac mesenchymal stem cells involved in tissue repair following myocardial infarction [28] as well as epicardium-derived cardiac fibroblasts [29]. In this regard, DDR2 may play an important role in cardiovascular regenerative medicine. VWF is also of major interest in tissue engineering, being a key component of platelet thrombus formation. Following vascular damage, multimers of VWF are recruited to the exposed subendothelial collagen and interact with the platelet glycoprotein GpIb/V/IX complex [30]. This initial association of platelets with the vessel wall allows subsequent binding of collagen receptors, integrin α_2_β_1_ and glycoprotein VI (GPVI), leading to strong platelet adhesion and activation [31]. VWF-mediated platelet recruitment might be useful in certain engineered devices, such as aortic stents, where thrombus deposition or tissue sealant activity is an important objective.

The synthetic sequence GPRGQOGV**Nle**GFO, with norleucine replacing methionine in VWFIII to avoid oxidation, has a similar affinity for DDR1 [32] and the A3 domain of VWF, and a higher affinity for DDR2 [33]. We therefore produced synthetic peptides containing this binding motif, flanked with 5 GPP triplets and with a 6-aminohexanoic acid linker on the N-terminus. This peptide is referred to as VWFIII_Nle_ in this study. We then end-stapled three VWFIII_Nle_ peptide strands to stabilise their subsequent triple-helical conformation. Many attempts to covalently link THP strands have been reported, through cysteine knots [34] or peptide elongation from di-lysine [35] but in our hands, these strategies gave poor results. Instead, we used a method described by Khew et al., where three peptide strands on solid support are covalently linked at their N-terminus using a short diglutamate-containing peptide (a tri-acid), generating a THP with an increased thermal stability and with a one-residue stagger between chains, as well as a single free N-terminus for derivatisation [36,37].

Next, end-stapled THPs were covalently linked to collagen matrices. Although THPs can be passively adsorbed by collagen fibers [21,38], we wished to prevent possible ligand elution from the collagen structure, from the perspective of long-term use in regenerative medicine. Our strategy uses a photoreactive group, diazirine, grafted on the N-terminus of the end-stapled THP, which can be readily activated by long wavelength UV light [[39], [40], [41]], producing a THP bearing a single reactive group. Thus, we have developed a controlled, efficient and rapid derivatisation of collagen matrices with pre-assembled and functional THPs.

In this study, EDC/NHS crosslinked collagen films were derivatised with VWFIII_Nle_-containing THPs and the effect on VWF and DDR2 activity was examined. First, we investigated restoration of the binding of DDR2 and VWF A3. Cell behavior beyond simple binding was also investigated through DDR2 phosphorylation triggered in transfected HEK293 and COS-7 cells by THP-derivatised collagen films. In parallel, we studied the ability of such films to support thrombus deposition from flowing blood, as well as promote Human Umbilical Vein Endothelial Cell (HUVEC) activity. Our data suggest that this approach will restore or enhance physiological function of collagen-based biomaterials.

## 2 Materials and methods

### 2.1 Peptide synthesis

#### 2.1.1 General procedure for peptide synthesis

9-Fluorenylmethoxycarbonyl (Fmoc) protected amino acids and *N*,*N*-Dimethylformamide (DMF) were supplied by AGTC Bioproducts (Hessle, UK). Fmoc-protected 6-Aminohexanoic acid (Ahx) was supplied by Merck (Darmstadt, Germany). All other amino acids and reagents were purchased from Sigma-Aldrich (Gillingham, UK). Peptides were synthesized using a Fmoc/*tert*-butyl solid phase strategy on a Liberty Blue™ microwave peptide synthesizer (CEM) at 0.1 mmol scale, using HCTU as coupling reagent and DIEA as base. (GPP)_5_GPRGQOGVNleGFO(GPP)_5_, Ahx-(GPP)_5_GPRGQOGVNleGFO(GPP)_5_ (VWFIII_Nle_), GPC(GPP)_5_GPOGPSGPRGQOGVMGFOGPKGNDGAO(GPP)_5_GPC (III-23) and GPC(GPP)_10_GPC (GPP10), were synthesized as C-terminal amides on Fmoc-Rink amide aminomethyl Tantagel resin (0.526 g, loading 0.19 mmol g^−1^, RAPP Polymere). The GFGEEG hexapeptide was synthesized as C-terminal acid on Fmoc-Gly-Wang (0.127 g, 0.79 mmol g^−1^, Novabiochem). Fmoc removal was performed with piperidine in DMF (20% v/v). Successive amino acid couplings and deprotection were carried out in DMF under microwave radiation. After peptide assembly, resin beads were washed with dichloromethane (DCM) twice, methanol (MeOH) twice and DCM.

#### 2.1.2 Peptide cleavage from the resin

Cleavage from the resin was performed over 2 h in 10 ml of a mixture of trifluoroacetic acid (TFA)/triisopropylsilane (TIS)/H_2_O 95/2.5/2.5 v/v/v. For III-23 and GPP10, both containing cysteine residues, 250 mg of dithiothreitol (DTT) was added to the cleavage mixture. The cleavage solution was concentrated and precipitated in 20 ml of cold diethyl ether. The white precipitate was filtered, washed with 10 ml of cold diethyl ether and redissolved in a H_2_O/acetonitrile (ACN) 95/5 v/v (0.1% TFA) mixture. The crude product was recovered after freeze drying and purified by preparative reverse-phase high performance liquid chromatography (RP-HPLC) on a Perkin Elmer LC200 system equipped with a 10 μm Eurospher II 100-10 C18 H (Knauer, Berlin, Germany) with a linear gradient of ACN 0.1% TFA in water 0.1% TFA. Purified compounds were characterized by matrix-assisted laser desorption ionization time-of-flight mass spectrometry (MALDI). Spectra of VWFIII_Nle_ and Diaz-ES-VWFIII_Nle_ can be found in Supporting Information S2.

#### 2.1.3 End-stapling of THPs

End-stapling of three strands of VWFIII_Nle_ was performed prior to cleavage from its solid support, loaded with 5 × 10^−5^ mol of synthesized peptide. 5 ml of a mixture of Fmoc-GFEEG-OH (4.5 mg, 5.6 × 10^−6^ mol), 1-[bis(dimethylamino)methylene]-1H-1,2,3-triazolo[4,5-b]pyridinium3-oxid-hexafluoro-phosphate (HATU, 6.3 mg, 1.67 × 10^−5^ mol), 1-hydroxybenzotriazole (2.6 mg, 1.67 × 10^−5^ mol) and *N*,*N*-diisopropylethylamine (DIEA, 5.8 μl, 3.3 × 10^−5^ mol) in DMF was added to the resin beads. The evolution of the reaction was followed by cleavage of 10 mg of dried resin as described above and analyzed by MALDI. Mass spectra show fragmented ions due to laser ionization as well as the characteristic −28 *m*/*z* adduct due to loss of N_2_. After 3 days at room temperature, no VWFIII_Nle_ were left unreacted and resin beads were washed with DCM twice, MeOH twice, and DCM. Removal of the Fmoc group was performed using 20% piperidine in DMF (v/v) for 45 min. The resin was further washed with DCM twice, MeOH twice and DCM.

#### 2.1.4 Transition temperature measurement

Peptides were solubilized in 900 μl of 10 mM phosphate buffer (with 150 mM NaCl) at a concentration of 2 mg/ml and the pH adjusted to 7.4. Peptide solutions were heated to 70 °C for 10 min to unfold the triple helix and kept at 4 °C overnight to refold. The melting temperature (T_m_) was measured by heating THP solutions from 8 °C to 80 °C at a ramp-rate of 0.5 °C/min in an Autopol III polarimeter. Optical rotation was measured every 15 s. T_m_ was determined by plotting the optical rotation and its first derivative against the temperature.

#### 2.1.5 Addition of diazirine on end-stapled THPs

Resin beads bearing end-stapled VWFIII_Nle_ (8.3 × 10^−6^ mol) were conditioned in 10 ml of dry DMF away from light for 5 min. DIEA (5.7 μl, 3.3 × 10^−5^ mol) and NHS-Diazirine (5.63 mg, 2.5 × 10^−5^ mol, Life Technologies) were added to the mixture. The reaction was left overnight at room temperature in the dark and the resin was washed with DCM twice, MeOH twice and DCM, to give the photoreactive peptide Diaz-ES-VWFIII_Nle_.

### 2.2 Cell lines and culture conditions

Human embryonic kidney (HEK) 293 cells and monkey COS-7 cells were from ATCC (Manassas, VA). Cells were cultured in Dulbecco's modified Eagle's medium/F12 nutrient mixture (Invitrogen) supplemented with 2 mM l-glutamine, 100 units/ml penicillin, 100 μg/ml streptomycin and 10% fetal bovine serum (FBS), at 37 °C with 5% CO_2_. Pooled Human Umbilical Vein Endothelial Cells (HUVECs) were purchased from Promocell (Heidelberg, Germany). Cells were cultured in Endothelial Cell Growth Medium 2 (EGM-2, Promocell) at 37 °C with 5% CO_2_.

#### 2.2.1 Transient transfection with DDR2-Flag

80–90% confluent COS-7 or Hek293 cells were seeded on 6-well plates for 24 h. Cos-7 cells were incubated for 4 h at 37 °C with 5% CO_2_ with a transfection solution containing 200 μl of OPTIMEM medium, 1.25 μg of DDR2-Flag DNA vector and 3 μl of Fugene per well, and were then left in fresh medium for 24 h at 37 °C with 5% CO_2_. Hek293 cells were transfected by calcium phosphate precipitation for 24 h at 37 °C with 5% CO_2_, as previously described [42]. 24 h after transfection, the cells were incubated in serum-free medium for a further 16 h, at 37 °C with 5% CO_2_.

### 2.3 Production of recombinant proteins

#### 2.3.1 DDR2-Fc preparation

Recombinant soluble protein comprising the entire DDR2 extracellular region, fused to the Fc-sequence of human IgG2, was produced in episomally-transfected HEK293-EBNA cells and purified by affinity chromatography as previously described [19,43].

#### 2.3.2 VWF A3-GST (glutathione S-transferase) preparation

A recombinant GST-tagged human VWF-A3 domain plasmid was obtained by cloning the VWF-A3 ORF into the bacterial expression vector pGEX-2T. To express VWF-A3 domain, a 100-ml overnight culture of transformants (Origami strain) was used to inoculate 1L of Luria broth containing 100 μg/ml ampicillin, 15 μg/ml kanamycin and 12.5 μh/ml tetracyclin. The culture was grown for 2 h at 37 °C and then induced at room temperature for 4 h with isopropyl β-d-thiogalacto-pyranoside (0.1 mM, Melford Laboratories, UK, #MB1008). Cells were harvested by centrifugation at 4500*g* for 20 min, and pellets were resuspended in 10 ml Dulbecco's phosphate-buffered saline, containing 1 tablet of protease inhibitor cocktail (Roche) and 5 mg of lysozyme (Fluka). Suspensions were sonicated and Triton X-100 was adjusted to 1% (v/v). Suspensions were incubated at room temperature for 15 min on a roller mixer and centrifuged at 18,000 g for 20 min and supernatants were pooled. The lysate was passed down a glutathione-agarose column equilibrated in Tris-buffered saline (20 mM Tris-HCl, pH 7.5, and 150 mM NaCl); the column was washed with 10 volumes of Tris-buffered saline containing 1 M NaCl and 1% (v/v) Triton X-100, and the GST-VWF A3 fusion protein was eluted with 10 mM glutathione reduced in 50 mM Tris-HCl (pH 8.0). The protein was then dialyzed against Tris-buffered saline and concentrated using a Microcon-3 (Amicon, Stonehouse, Gloucestershire, UK). The protein was checked for purity and degradation using 10% SDS-PAGE and Western blotting. Nitrocellulose blots were probed with horseradish peroxidase-conjugated anti-glutathione S-transferase polyclonal antibody (GE Healthcare).

### 2.4 Preparation of EDC/NHS crosslinked collagen films

Collagen films were prepared as previously described [8,15]. Briefly, bovine Achilles tendon collagen type I (Sigma, #4387, Gillingham, UK) was suspended in 0.05 M AcOH at a concentration of 0.5% w/v and left to swell overnight at 4 °C. The mixture was homogenized using an Ultraturrax VD125 blender for 20 min at 13500 rpm. It was then centrifuged to remove air bubbles 5 min at 2500 rpm (Hermle Z300, Labortechnik, Germany) and further homogenized 10 min at 13500 rpm. After centrifuging again 5 min at 2500 rpm, the resulting slurry was left overnight at room temperature before use.

#### 2.4.1 Coating on 96-well plates and 8-well glass slides

Immulon-2 HB 96-well plates or BD Falcon 8-chamber culture slides were coated with 100 μl of 0.5% w/v collagen slurry and left to dry 3 days in a fume hood. Film crosslinking was performed using 200 μl of a mixture of EDC and NHS in 70% EtOH for 2 h at room temperature. Standard crosslinking conditions (referred to as 100% crosslinking) used a 5/2/1 EDC/NHS/collagen molar ratio (1.115 g EDC, 0.276 g NHS per gram of collagen). Different degrees of crosslinking were achieved by using different concentrations of EDC and NHS, with a EDC/NHS/collagen molar ratio ranging from 0.05/0.02/1 (for 1% crosslinking) to 5/2/1 (for 100% crosslinking). Wells were then washed with EtOH three times for 10 min and deionized water three times for 10 min. Plates were left to dry for 3 days in a fume hood before use.

#### 2.4.2 Coating on 12-well plates and glass cover slips

Nunc 12-well plates or 13 mm glass coverslips were coated with 400 μl of 0.5% w/v collagen slurry and left to dry three days in a fume hood. Collagen films were then crosslinked to 100% using an EDC and NHS mixture at a 5/2/1 EDC/NHS/collagen molar ratio (1.115 g EDC, 0.276 g NHS per gram of collagen) in EtOH 70% for 2 h at room temperature. Wells were then washed with EtOH three times for 10 min and deionized water three times for 10 min. Plates were left to dry for 3 days in a fume hood before use.

### 2.5 DDR2 and VWF A3 binding to crosslinked collagen films

Collagen films prepared on 96-well plates were crosslinked with EDC and NHS to degrees of 0%, 1%, 10%, 25%, 50% and 100%. Wells were blocked with 200 μl of PBS 5% BSA during 45 min. Wells were then washed three times with 200 μl of PBS 0.1% BSA. 100 μl of the recombinant protein in PBS 0.1% BSA at 5 μg/ml of VWF A3 fused to GST, or 1 μg/ml of DDR2 fused to Fc with 0.05% Tween-20, were incubated on collagen films for 2 h at room temperature. After washing wells three times with 200 μl of PBS 0.1% BSA, 100 μl of anti-GST (for VWF A3) or anti-Fc (for DDR2) HRP-conjugated antibodies diluted 1:10000 were added to wells for 1 h at room temperature. After washing wells four times with 200 μl of PBS 0.1% BSA, 100 μl of TMB substrate was added. The reaction was quenched with 100 μl of 2.5 M sulfuric acid and absorbance at 450 nm or 370 nm was measured [44].

### 2.6 Collagen film derivatisation with THPs

VWFIII_Nle_ or Diaz-ES-VWFIII_Nle_ were diluted to 5 μg/ml in PBS (pH 7.4) from a 5 mg/ml stock solution in 0.01 M AcOH. 100 μl (for experiments on 96-well plates or 8-well glass slides) or 400 μl (for experiments on glass coverslips or 12-well plates) of peptide solution were added to collagen films and incubated for 30 min in the dark at room temperature. Collagen films were then placed under a long-wavelength UV lamp (Blak-Ray B100AP, 365 nm wavelength) for 5 min. Wells were then washed with 200 μl of citrate buffer (pH 3) 0.1% BSA, 6 × 2 min, and 200 μl of PBS 0.1% BSA, 6 × 2 min.

### 2.7 VWF A3-GST binding to derivatised collagen films

100% EDC/NHS crosslinked collagen films in 96-well plates were derivatised with Diaz-ES-VWFIII_Nle_ or VWFIII_Nle_ as described above. Wells were blocked with 200 μl of PBS containing 50 mg/ml of BSA during 45 min. Wells were then washed three times with 200 μl of PBS 0.1% BSA. 100 μl of VWF A3 fused to GST (VWF-A3-GST) at 5 μg/ml in PBS 0.1% BSA, or DDR2 fused to Fc (DDR2-Fc) at 1 μg/ml in PBS 0.1% BSA and 0.05% Tween-20 were added to collagen films for 2 h at room temperature. After washing wells three times with 200 μl of PBS 0.1% BSA, 100 μl of anti-GST or anti-Fc HRP-conjugated antibody diluted 1:10000 were added to wells for 1 h at room temperature. After washing wells four times with 200 μl of PBS 0.1% BSA, 100 μl of TMB substrate were added. The reaction was quenched with 100 μl of 2.5 M sulfuric acid and absorbance at 450 nm was measured [44].

### 2.8 DDR2 activation on derivatised collagen films

#### 2.8.1 By immunofluorescence

COS-7 cells were washed once with PBS, detached using 1 ml/well of trypsin/EDTA solution for 3 min at 37 °C with 5% CO_2_ and diluted in 5 ml of complete medium per well. Cells were centrifuged for 5 min at 200 *g* and resuspended in 2 ml per well of serum free medium. 500 μl of cells in suspension were added to 13 mm cover slips coated with derivatised collagen films (prepared as described above) for 2 h at 37 °C with 5% CO_2_. Cells were then fixed with 500 μl of 4% paraformaldehyde in PBS for 15 min at room temperature. After three washes with 500 μl of PBS, cells were permeabilised for 5 min with 0.5% of Triton X-100 in PBS. Cells were blocked with filtered PBS 5% BSA for 1 h. After three washes in PBS, cover slips were incubated with 30 μl of 1:100 mouse M2 anti-Flag antibody (Sigma-Aldrich) and 1:500 rabbit anti-phospho-DDR2 (Tyr740, from R&D Systems) antibody for 1 h. After three further washes in PBS, cover slips were incubated with 30 μl of 1:500 anti-mouse Alexa Fluor 488 and 1:500 anti-rabbit Alexa Fluor 555 antibody (Life Technologies) for 1 h. Cover slips were washed three times with PBS and once with water, and mounted on glass slides with 8 μl of Prolong Gold anti-fade media (Life Technologies). Glass slides were left to dry 24 h before use.

#### 2.8.2 By western blot

After removing the media, HEK293 cells were detached by pipetting up and down 1 ml per well of serum free media and diluted further with an extra 1 ml per well of serum-free media. 1 ml of cells in suspension was added to derivatised collagen films coated on 12-well plates (prepared as described above) for 90 min at 37 °C with 5% CO_2_. Cell were lysed with 80 μl of lysis buffer (150 mM NaCl, 50 mM Tris pH 7.4, 1 mM EDTA, 1 mM PMSF, 50 μg/ml aprotinin, 5 mM NaF and 1 mM NaVO_3_) for 20 min on ice and kept at −80 °C overnight. Cell lysates and a blue pre-stained protein ladder (Broad range 11–190 kDa, NEB) were separated on a SDS-PAGE 4–12% gel and transferred to a PVDF membrane. Membranes were blocked with Odyssey blocking buffer (LICOR Bioscience), probed with both anti-Flag 1:500 and anti-pY740 1:1000 antibodies overnight at 4 °C, washed with blocking buffer 3 × 10 min, probed with anti-mouse 800CW (LICOR Bioscience) and anti-rabbit 680RD (LICOR Bioscience) for 1 h, and washed 3 × 5 min. Signals were acquired and quantified using the Odyssey instrument (LICOR Bioscience).

### 2.9 Platelet flow studies

100% EDC/NHS crosslinked collagen films coated on 8-well glass slides were derivatised with Diaz-ES-VWFIII_Nle_ as described above and soaked with Fc buffer (NaCl 136 mM, KCl 2.7 mM, HEPES 5 mM, glucose 10 mM, MgCl_2_ 2 mM, CaCl_2_ 2 mM, BSA 1% and Heparin 100 U/unit). Blood from healthy volunteers free from medication for 2 weeks was collected into tubes containing 40 μM D-phenylalanyl-prolyl-arginyl chloromethyl ketone (PPACK) and supplemented every hour with 10 μM PPACK. Before use, blood was incubated with 1 μM 3,3-dihexyloxacarbocyanine iodide (DiOC_6_) for 15 min. 1.2 ml of whole blood was perfused at a shear rate of 1000 s^−1^ on collagen-coated glass slides using a pulse-free pump at room temperature. Collagen films were washed with 500 μl of Fc buffer, fixed with 4% paraformaldehyde during 15 min at room temperature and washed with a further 500 μl of Fc buffer. Mounting media was added, cover slips were mounted on glass slides and were left to dry 24 h before use.

### 2.10 HUVECs acetylated low-density lipoprotein (Ac-LDL) uptake

70–80% confluent HUVECs were serum starved for 2 h in EBM-2 Basal Medium (Promocell) with 0.5% FBS at 37 °C with 5% CO_2_ and detached with 5 ml of trypLE (Gibco) for 5 min at room temperature. Cells were diluted in 10 ml of PBS with 10% FBS, centrifuged for 5 min at 230 g and resuspended in EGM-2 to reach a concentration of 100 000 cells/ml. 500 μl of cell suspension with 2.5 μl of Ac-LDL-Dil (Thermo Fisher) were incubated on 100% EDC/NHS crosslinked collagen films coated on 12-well plates were derivatised with Diaz-ES-VWFIII_Nle_ as described above. Cells were left 24 h at 37 °C with 5% CO_2,_ fixed with 4% paraformaldehyde for 15 min at room temperature and washed three times with PBS. Cell nuclei were stained with Hoechst 33342 nucleic acid stain (Thermo Fisher) diluted 1:2000 in PBS for 15 min at room temperature. Cells were washed twice in PBS before imaging.

### 2.11 Image acquisition

Fluorescent images were obtained using an Olympus FV300 laser-scanning confocal microscope. For DDR2 activation study, fluorescence intensity of Alexa Fluor 555 and Alexa Fluor 488 was quantified on 10 randomly selected fields of view, using identical microscope settings between conditions and experiments. On each repeat, ratio of fluorescence intensity of Alexa Fluor 555/Alexa Fluor 488 was calculated and normalized to the value obtained on non-crosslinked collagen films. For platelet flow studies, percentage of surface covered by platelets was carried out using a method described previously [21]. Briefly, vertical sequences (Z-stacks) of images of a field of view encompassing the collagen films and the entire thrombus were acquired with 0.69 μm steps. For each experiment, Z-stacks from 6 randomly selected fields of view were exported to ImageJ 1.46 (National Institutes of Health) for analysis. Surface coverage of platelets was measured from the collagen film plane as percentage of the field area. For LDL uptake studies, images were obtained using a Leica DM6000 FS fluorescence microscope. Fluorescence intensity of 1,1′-dioctadecyl-3,3,3′,3′-tetramethylindocarbocyanine perchlorate (Dil) and Hoechst 33342 was quantified on 10 randomly selected fields of view, using identical microscope settings between conditions.

### 2.12 Statistical analysis

Values shown are mean ± SEM. Mean values were compared using Prism software (GraphPad, San Diego) and 1-way ANOVA or 2-way ANOVA. Tukey's post hoc tests were performed for comparison between conditions, and in Figure legends **** denotes p < 0.0001, *** denotes p < 0.001, ** denotes p < 0.01 and * denotes p < 0.05.

## 3 Results

### 3.1 VWF A3 and DDR2 binding to crosslinked collagen films

**Fig. 1:**
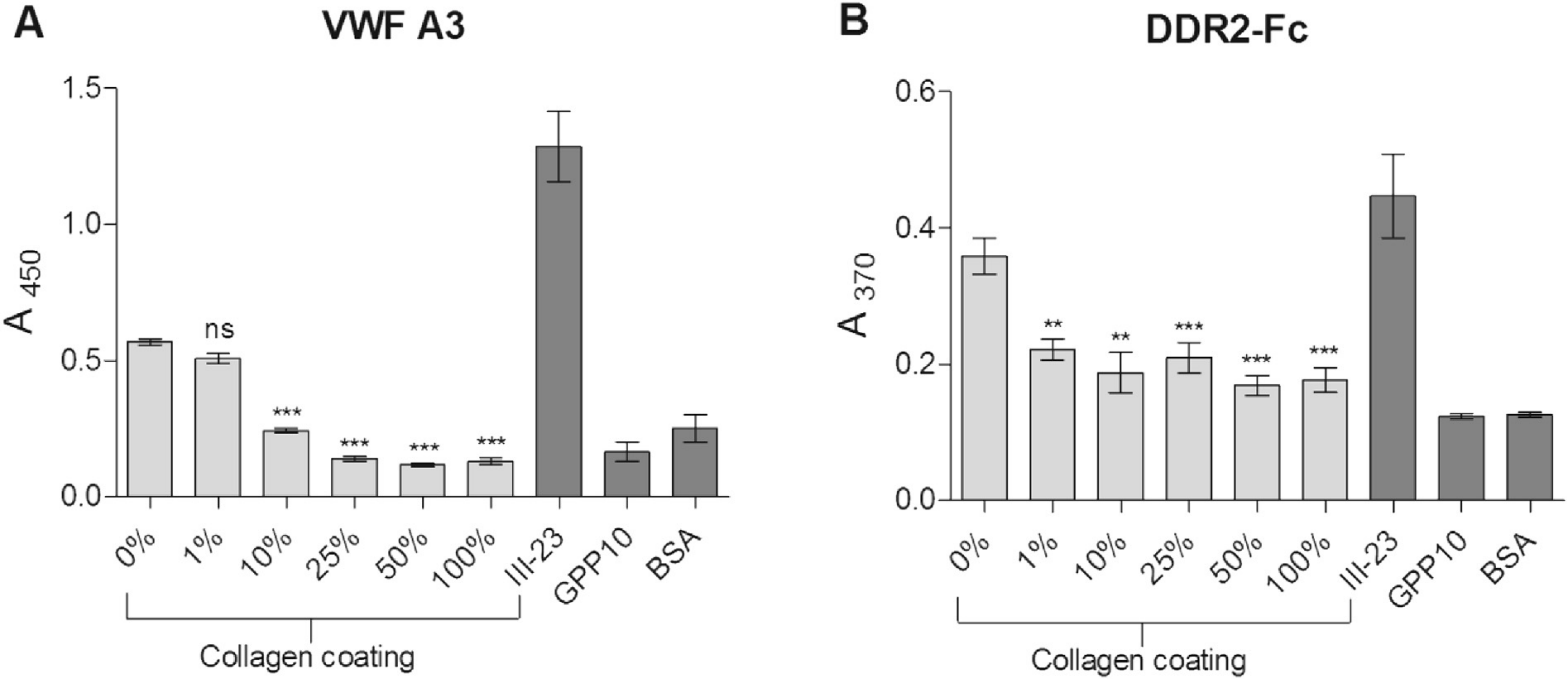
VWF A3 and DDR2 binding to crosslinked collagen films. Wells were coated with collagen films and subsequently treated with various concentrations of EDC and NHS in 70% EtOH, ranging from 1% to 100% of the standard concentration (100%) for 2 h at room temperature. VWF-A3-GST (A) at 5 μg/ml for 1 h or DDR2-Fc (B) at 1 μg/ml for 2 h were incubated on crosslinked collagen films at room temperature, and detected with anti-GST and anti-Fc antibodies respectively. Additional wells without collagen were coated with III-23 or GPP10 for positive and negative control respectively at 10 μg/ml in AcOH 0.01 M overnight. These graphs show mean values with standard error of pooled results of three independent data sets (n = 3 for each condition within each experiment; 1-way ANOVA, p < 0.01). Overall, VWF and DDR2 binding decreased as the degree of film crosslinking increased. Statistical significance is shown compared to 0% EDC/NHS for all other EDC/NHS concentrations.

DDR2 and VWF A3 binding was evaluated on collagen films prepared in 96-well plates and crosslinked with EDC/NHS to different degrees ranging from 0% to 100% (Fig. 1). Additional wells without collagen were coated with either III-23, a peptide with high affinity for VWF A3 and DDR2 and used here as positive control [23], or GPP10, a THP with no active sequence, as a negative control. VWF-A3-GST binding was gradually diminished by increasing EDC/NHS concentration for crosslinking degrees ranging from 0% (A_450_ = 0.57 ± 0.03) to 50% (A_450_ = 0.12 ± 0.02, p < 0.001) and remaining low at 100% (A_450_ = 0.13 ± 0.03, p < 0.001) (Fig. 1 A). A similar trend was observed with the DDR2-Fc fusion protein (Fig. 1 B). However, we observed the formation of a precipitate on non-crosslinked films upon addition of sulfuric acid, resulting in flawed absorbance values at 450 nm. Instead, A_370_ was read before addition of sulfuric acid. DDR2-Fc binding decreased significantly as the degree of crosslinking rose from 0% (A_370_ = 0.32 ± 0.03) to 100% (A_370_ = 0.18 ± 0.05, p < 0.001). Overall, those results highlight the loss in protein adhesion, and thus the need to restore receptor activity to collagen films treated with EDC/NHS in the standard 100% crosslinking conditions.

### 3.2 Synthesis of photoreactive THPs

**Fig. 2:**
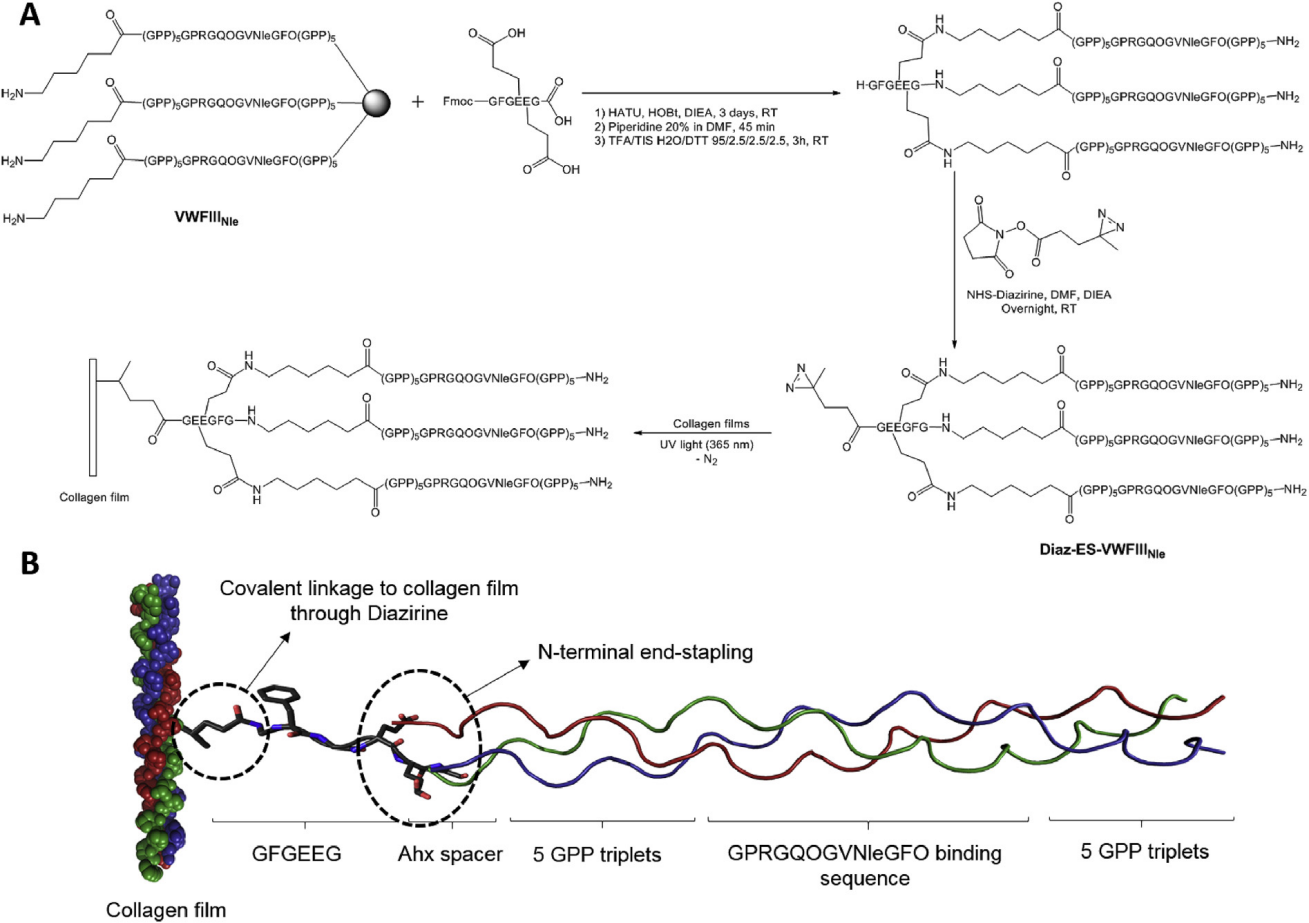
A) Synthetic pathway for the synthesis of end-stapled THPs containing the active sequence GPRGQOGVNleGFO. VWFIII_Nle_ represents the peptide Ahx-(GPP)_5_GPRGQOGVNleGFO(GPP)_5_. B) Scheme of the THP construct covalently linked on crosslinked collagen film following UV treatment. Diazirine carried by Diaz-ES-VWFIII_Nle_ undergoes UV activation, forming a covalent bond with the collagen film. It thus links the collagen film to the GFGEEG motif, followed by three strands composed of the Ahx spacer and the active sequence GPRGQOGVNleGFO flanked by five GPP triplets.

Fig. 2 A shows the synthetic scheme of THPs and its linkage to collagen films. Peptide strands were first assembled using standard Fmoc/*tert-*Butyl chemistry using microwave irradiation for coupling and deprotection steps. In this study, the DDR2- and VWF-binding sequence GPRGQOGVNleGFO was incorporated into a host peptide containing five GPP triplets on both C- and N-termini. Peptides containing this sequence have been previously used in our lab with additional GPC triplets [18,21,23]. An additional Ahx linker was added to the N-terminus of the peptide, to give more flexibility and accessibility to the N-terminal primary amine and to allow better reaction with carboxylic acid during the following end-stapling step. The N-terminal end-stapling of peptide strands within a triple helix and addition of diazirine were carried out as described in Section 2.1. The diazirine photoreactive group was chosen for its sensitivity to long UV wavelength (330–370 nm) which does not damage or alter the collagen substrate. Diazirine also has better stability in visible light than most photo-activatable groups while having a quicker activation time (half-life of 4 min at 8 W), allowing highly efficient linkage to a substrate under UV at 360 nm [45]. Cleavage of the photoreactive end-stapled THP was performed using a TFA/TIS/H_2_O cocktail. The end-stapled THP was then precipitated in cold diethyl ether, freeze dried and purified by semi-preparative HPLC away from light. To evaluate the effect of end-stapling on the triple helix stability, the transition temperatures (Tm) of peptides before and after the reaction were measured by polarimetry (see Supporting Information S2). Tm rose from 45 °C for VWFIII_Nle_ to 51 °C for Diaz-ES-VWFIII_Nle_. End-stapling thus resulted in an increase of 6 °C in the thermal stability of the triple helix.

### 3.3 Collagen film derivatisation with THPs

Peptides were pre-incubated on collagen films for 30 min [21]. Passively adsorbed THPs were then covalently attached to collagen films by UV treatment. In our previous study, conditions for collagen film derivatisation were optimised and the UV treatment time was set to 5 min with a peptide concentration of 5 μg/ml [15]. Upon UV treatment, nitrogen is released as an inert by-product as the diazirine moiety forms a highly reactive carbene intermediate which can readily react to form a covalent bond between the THP and the carbon skeleton of collagen (Fig. 2 B). In subsequent experiments, collagen films were either protected from light so that only passively adsorbed peptides remained after washing steps, or treated with UV light, where both passively adsorbed and covalently linked peptides were present.

### 3.4 VWF A3 binding to derivatised 100% crosslinked films

**Fig. 3:**
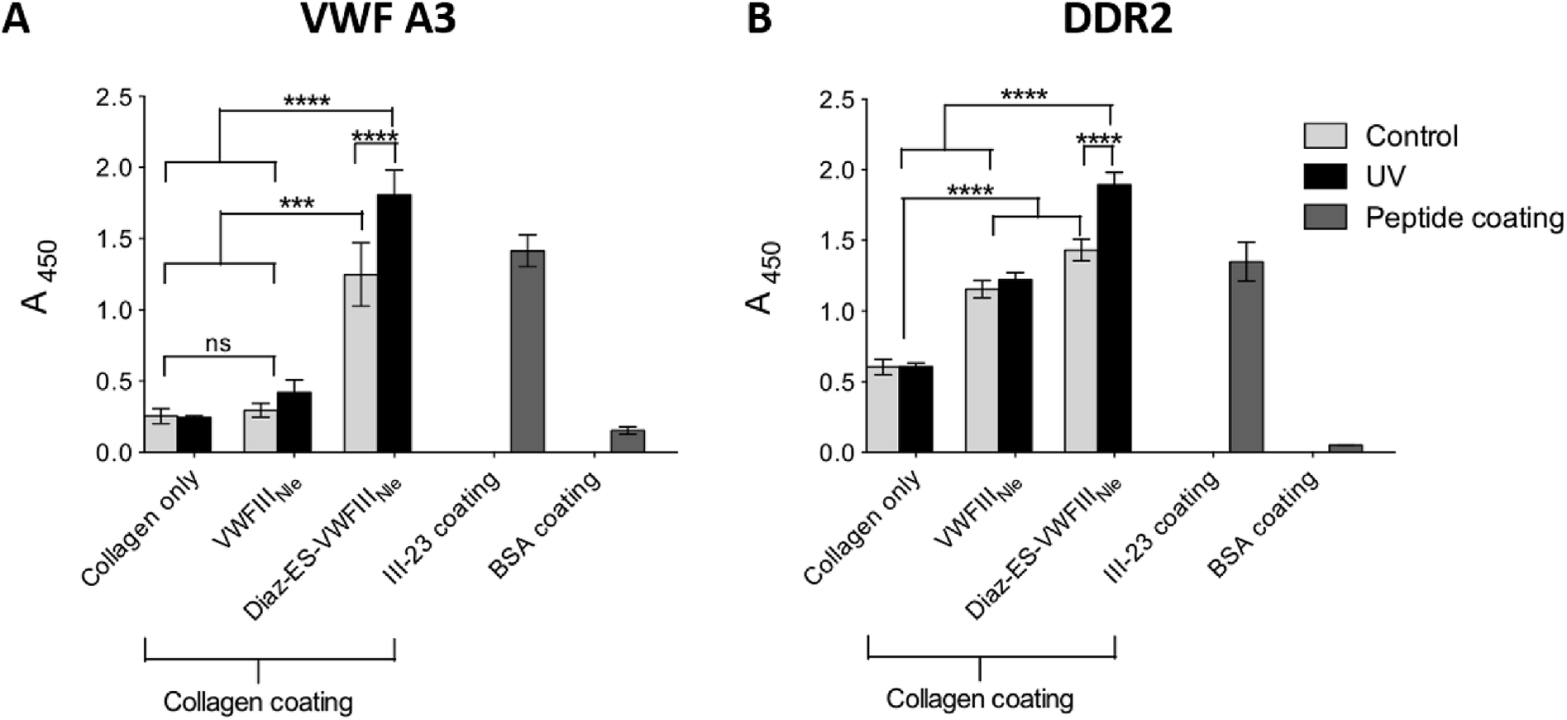
VWF A3 and DDR2 binding to collagen films derivatised with GRPGQOGVNleGFO. Wells were coated with 100% EDC/NHS crosslinked collagen films. Diaz-ES-VWFIII_Nle_ or VWFIII_Nle_ were added to films at 5 μg/ml and left in the dark or exposed to UV light for 5 min. Controls included wells coated with III-23 or BSA. VWF A3-GST (A) or DDR2-Fc (B) fusions were incubated on collagen films at 5 μg/ml for 1 h or 1 μg/ml for 2 h at room temperature, and detected using anti-GST and anti-Fc antibodies respectively. These graphs show pooled results of three independent data sets (n = 3 for each condition within each experiment). Diaz-ES-VWFIII_Nle_ followed by UV treatment led to a significant increase in both VWF A3-GST and DDR2-Fc binding (2-way ANOVA, p < 0.001).

100% crosslinked collagen films were treated with various THPs and the binding of VWF A3 was evaluated (Fig. 3 A). Non-derivatised films supported low binding, both with (A_450_ = 0.24 ± 0.01) and without UV treatment (A_450_ = 0.26 ± 0.05), close to that of BSA coatings (A_450_ = 0.15 ± 0.02). Binding after incubation with non-photoreactive peptides remained low, both with UV (A_450_ = 0.42 ± 0.09) and without UV (A_450_ = 0.30 ± 0.05). However, collagen film derivatised with photoreactive Diaz-ES-VWFIII_Nle_ led to a dramatic increase in binding of VWF A3. Most importantly, binding following incubation with Diaz-ES-VWFIII_Nle_ and UV treatment led to a seven-fold increase in absorbance (A_450_ = 1.80 ± 0.17), higher than wells coated with III-23 (A_450_ = 1.42 ± 0.11). No UV treatment also resulted in relatively high affinity (A_450_ = 1.25 ± 0.22), probably due to passive absorption of this particular peptide. Tukey's post hoc tests on three independent repeats showed significant increase in binding with Diaz-ES-VWFIII_Nle_ followed by UV treatment compared to other conditions on collagen films (p < 0.0001).

### 3.5 DDR2 binding to derivatised 100% crosslinked films

100% crosslinked collagen films were derivatised with THPs and their ability to bind DDR2-Fc fusion protein was investigated (Fig. 3 B). Additional wells were coated with BSA or peptide III-23 for negative and positive controls respectively. Once again, binding to collagen films alone was low regardless of UV treatment (A_450_ = 0.61 ± 0.03) but higher than on BSA coating (A_450_ < 0.2), supporting the results obtained above. Film derivatisation with Diaz-ES-VWFIII_Nle_ after UV treatment led to a significant three-fold increase in DDR2-Fc binding (A_450_ = 1.90 ± 0.09, p < 0.0001). All other peptide treatments supported intermediate binding (A_450_ between 1.16 and 1.43), significantly different from both untreated and UV/Diaz-ES-VWFIII_Nle_ treated films, but not from each other. We assume that passive adsorption of peptides was responsible for the DDR2 binding observed without diazirine or UV treatment, despite numerous washing steps.

### 3.6 DDR2 activation on derivatised 100% crosslinked films detected by immunofluorescence

**Fig. 4:**
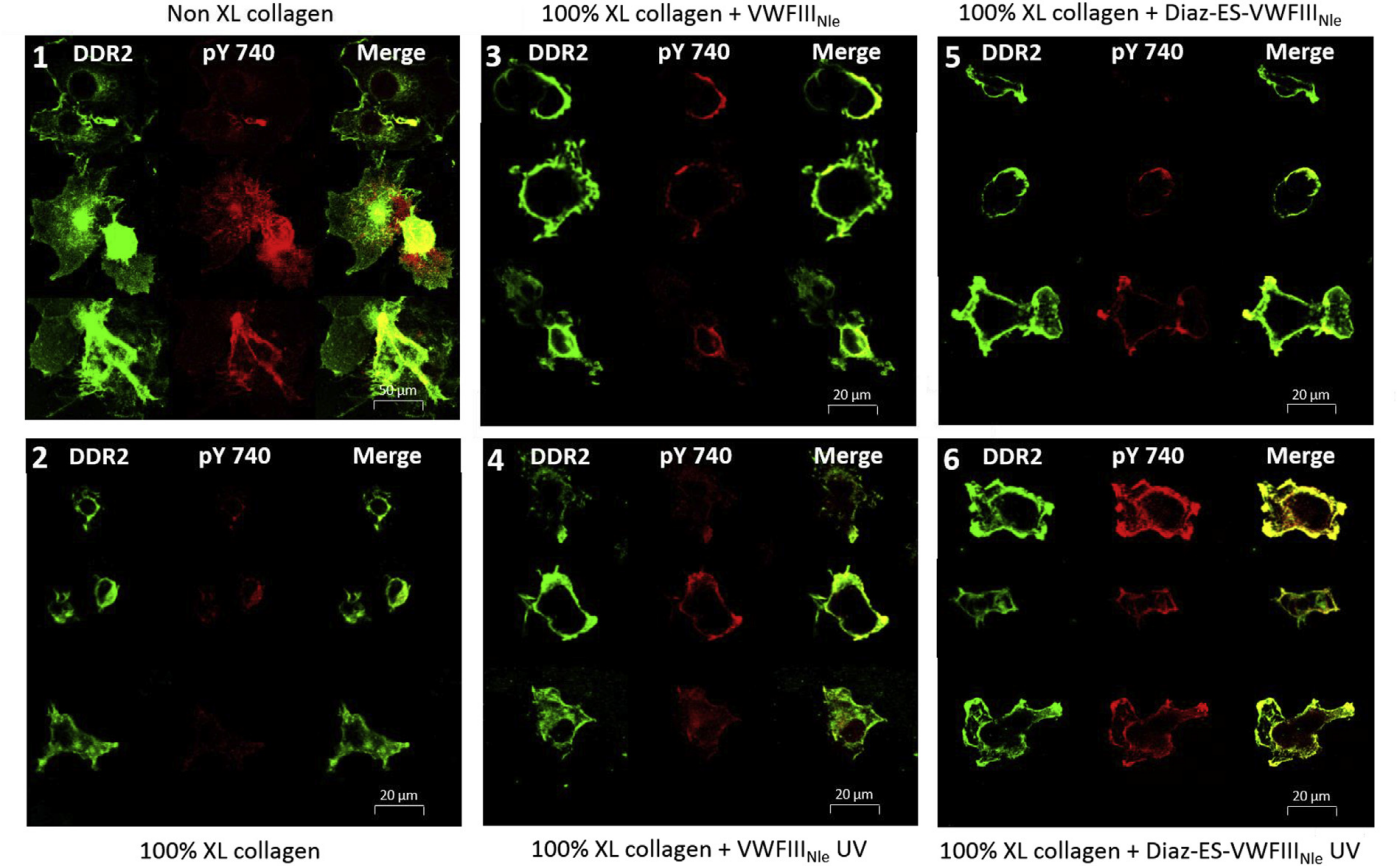
Confocal images of DDR2 expressing COS-7 cells on derivatised collagen films. Where indicated, peptides were incubated at 5 μg/ml and exposed to UV light for 5 min. COS-7 cells, transiently transfected with DDR2-Flag, were incubated on films for 2 h. Collagen films were non-crosslinked (1); or 100% crosslinked with no peptides (2); with VWFIII_Nle_ no UV (3); with VWFIII_Nle_ after UV (4); with Diaz-ES-VWFIII_Nle_ no UV (5); or with Diaz-ES-VWFIII_Nle_ after UV (6). DDR2-Flag was detected with anti-Flag and Alexa Fluor 488 antibody. DDR2 phosphorylation on tyrosine 740 was detected with pY740 and Alexa Fluor 555 antibody. Representative cells taken from five different experiments are shown for each conditions.

**Fig. 5:**
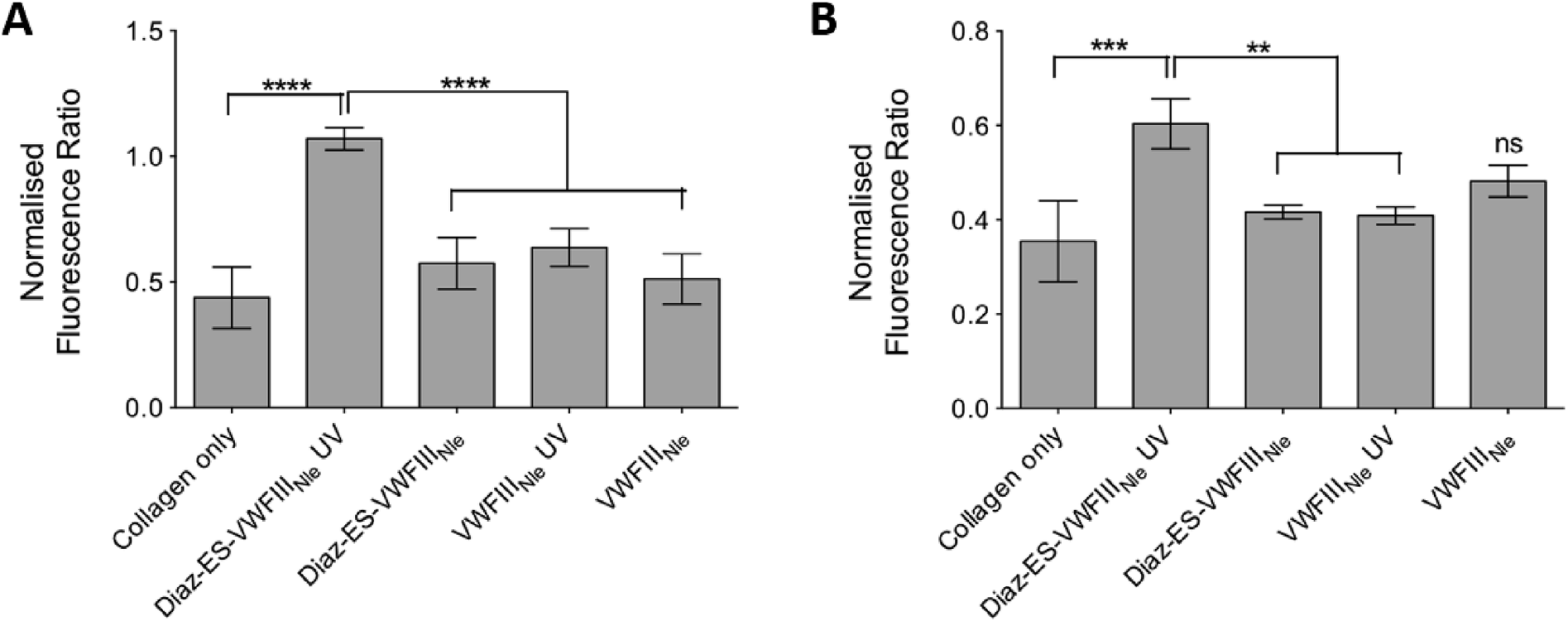
Collagen films were crosslinked with 100% EDC/NHS, incubated with Diaz-ES-VWFIII_Nle_ or VWFIII_Nle_ at 5 μg/ml and left in the dark or exposed to UV light for 5 min. COS-7 or HEK-293 cells, transiently transfected with DDR2-Flag, were incubated on films. For each condition, fluorescence intensity ratio of DDR2 phosphorylation over DDR2-Flag was measured. Y axis shows the ratio normalized to the value obtained with non-crosslinked collagen film. (A) Fluorescence intensity measured in COS-7 cells by confocal microscopy on 10 randomly selected fields of view for each conditions within each repeat. This graph shows pooled results from five independent data sets (n = 10 for each condition within each experiment; 1-way ANOVA, p < 0.0001). (B) Fluorescence intensity of DDR2 extracted from HEK293 cells and measured on western blot membrane. This graph shows pooled results from three independent data sets. Diaz-ES-VWFIII_Nle_ followed by UV treatment led to a significant increase in DDR2 phosphorylation, as measured by immunofluorescence or western blot (1-way ANOVA, p < 0.001).

**Fig. 6:**
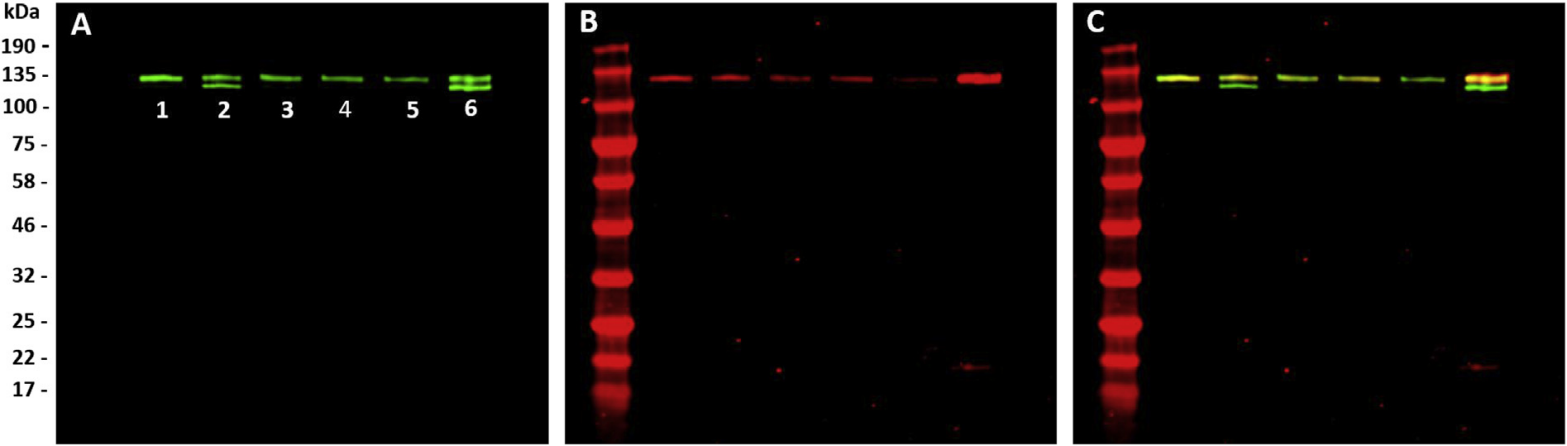
Western blot of HEK-293 cell lysates transfected with DDR2-Flag and incubated for 90 min on 100% EDC/NHS crosslinked films derivatised with peptides at 5 μg/ml and left in the dark or exposed to UV light for 5 min. Collagen films were non-crosslinked (Lane 6); or 100% crosslinked with no peptides (Lane 5); with VWFIII_Nle_ no UV (Lane 4); with VWFIII_Nle_ after UV (Lane 3); with Diaz-ES-VWFIII_Nle_ no UV (Lane 2); or with Diaz-ES-VWFIII_Nle_ after UV (Lane 1). DDR2-Flag and DDR2 phosphorylation were detected on a single membrane. (A) DDR2-Flag staining with anti-Flag and anti-mouse 800CW detected at 800 nm; (B) DDR2 phosphorylation staining with pY740 and anti-rabbit 680 RD detected at 700 nm; (C) Merge. Western blots show co-localization of 700 nm and 800 nm fluorescence on a single membrane between 125 and 130 kDa, confirming that antibodies used in our experiments target specifically DDR2-Flag and DDR2 phosphorylation as expected.

The DDR2 expression in COS-7 cells is reported here using the C-terminal Flag tag of the construct, detected with an anti-Flag and Alexa Fluor 488-conjugated secondary antibody, marking the receptors in green, as shown in Fig. 4 and labelled DDR2. DDR2 activation (autophosphorylation) was detected using a primary antibody that only recognizes phosphorylated DDR2, followed by a secondary Alexa fluor 555 antibody, marking the activated DDR2 receptor in red (Fig. 4, pY740). We first verified that DDR2-Flag in COS-7 cells was activated on non-crosslinked collagen films (Fig. 4-1). In contrast to this situation, experiments carried out on 100% crosslinked films showed only limited DDR2 phosphorylation despite adequate DDR2 expression (Fig. 4-2). The addition of VWFIII_Nle_ (Figs. 4-3 and 4-4) or Diaz-ES-VWFIII_Nle_ without UV (Fig. 4-5) led to moderate activation on some cells. With Diaz-ES-VWFIII_Nle_ and UV treatment, however, DDR2 phosphorylation (Fig. 4-6) reached levels similar to that observed on non-crosslinked films (Fig. 4-1). All fluorescence due to tyrosine 740 phosphorylation was co-localized with DDR2, indicating that our system only detects fluorescence due to DDR2 phosphorylation as expected. We then noticed that, in contrast to native collagen, cells on 100% crosslinked films remained regular in outline, rounded and rarely spread well on the collagen surface, highlighting the need for integrins to support cell spreading on collagen films, as described in our previous study [15].

DDR2 expression and phosphorylation in COS-7 cells was quantified on 10 randomly selected fields of view for each condition and in each of 5 independent repeats (Fig. 5 A). The ratio of integrated fluorescence intensity of phosphorylated DDR2 tyrosine 740 (Alexa Fluor 555) to DDR2-Flag fluorescence (Alexa Fluor 488) reflects the proportion of DDR2 that is activated, regardless of DDR2 expression level. Within each experimental repeat, the integrated intensity ratio for each condition was normalized to the ratio calculated for non-crosslinked collagen films, thus eliminating variation in antibody binding efficiency from one repeat to another. On non-crosslinked films, Alexa Fluor 555 fluorescence (pY740) reached 74% of Alexa Fluor 488 fluorescence (total DDR2), dropping to 32% (0.43 ± 0.27 times non-crosslinked value) after EDC/NHS treatment. A moderate increase in integrated intensity ratio was detected in the presence of Diaz-ES-VWFIII_Nle_ without UV treatment (42%, 0.57 ± 0.23 times non-crosslinked value), VWFIII_Nle_ with UV (47%, 0.64 ± 0.17 times non-crosslinked value) and VWFIII_Nle_ without UV (38%, 0.51 ± 0.22 times non-crosslinked value). Diaz-ES-VWFIII_Nle_ after UV treatment, however, led to a significant increase in DDR2 phosphorylation (79%, 1.07 ± 0.10 times non-crosslinked value, p < 0.0001).

### 3.7 DDR2 activation on derivatised 100% crosslinked films detected by western blot

As for immunofluorescence assays, HEK293 cells were transfected with a DDR2-Flag construct, and DDR2 expression and phosphorylation was assessed using anti-Flag and pY740 antibodies. Both antibodies were incubated on a single membrane to allow better comparison and revealed with near-infrared fluorescent antibodies (at 800 nm for DDR2 and 700 nm for phosphorylated DDR2, Fig. 6). DDR2 often appears as a mixture of up to four bands between 125 kDa and 130 kDa, corresponding to different levels of glycosylation [22,43,46]. No other bands outside this range was observed in our case, confirming that the antibodies used both for immunofluorescence and western blotting were indeed specific to DDR2.

In agreement with immunofluorescence results, strong DDR2 activation was observed on non-crosslinked collagen films (Fig. 6 B Lane 6) while only low levels of phosphorylation were detected on 100% crosslinked films (Fig. 6 B Lane 5). Moreover, VWFIII_Nle_ (Fig. 6 B Lane 4 and 6 B Lane 3) or Diaz-ES-VWFIII_Nle_ without UV (Fig. 6 B Lane 2) helped restore only partial DDR2 phosphorylation, whereas UV-bound Diaz-ES-VWFIII_Nle_ allowed almost complete DDR2 activation (Fig. 6 B Lane 1). The fluorescent intensity of bands from three independent experiments for DDR2 and DDR2 phosphorylation were quantified as described in section 2.8. DDR2 phosphorylation on 100% crosslinked films accounted only for 0.35 ± 0.09 times the phosphorylation level observed on non-crosslinked films, rising to 0.60 ± 0.13 times in the presence of covalently linked Diaz-ES-VWFIII_Nle_ (p < 0.001, Fig. 5 B). For other conditions, this value reached 0.42 ± 0.04 (p < 0.01), 0.41 ± 0.05 (p < 0.01) and 0.48 ± 0.08 (non significant) for Diaz-ES-VWFIII_Nle_ without UV, VWFIII_Nle_ with UV and VWFIII_Nle_ without UV respectively.

### 3.8 Platelet flow study on derivatised 100% crosslinked films

**Fig. 7:**
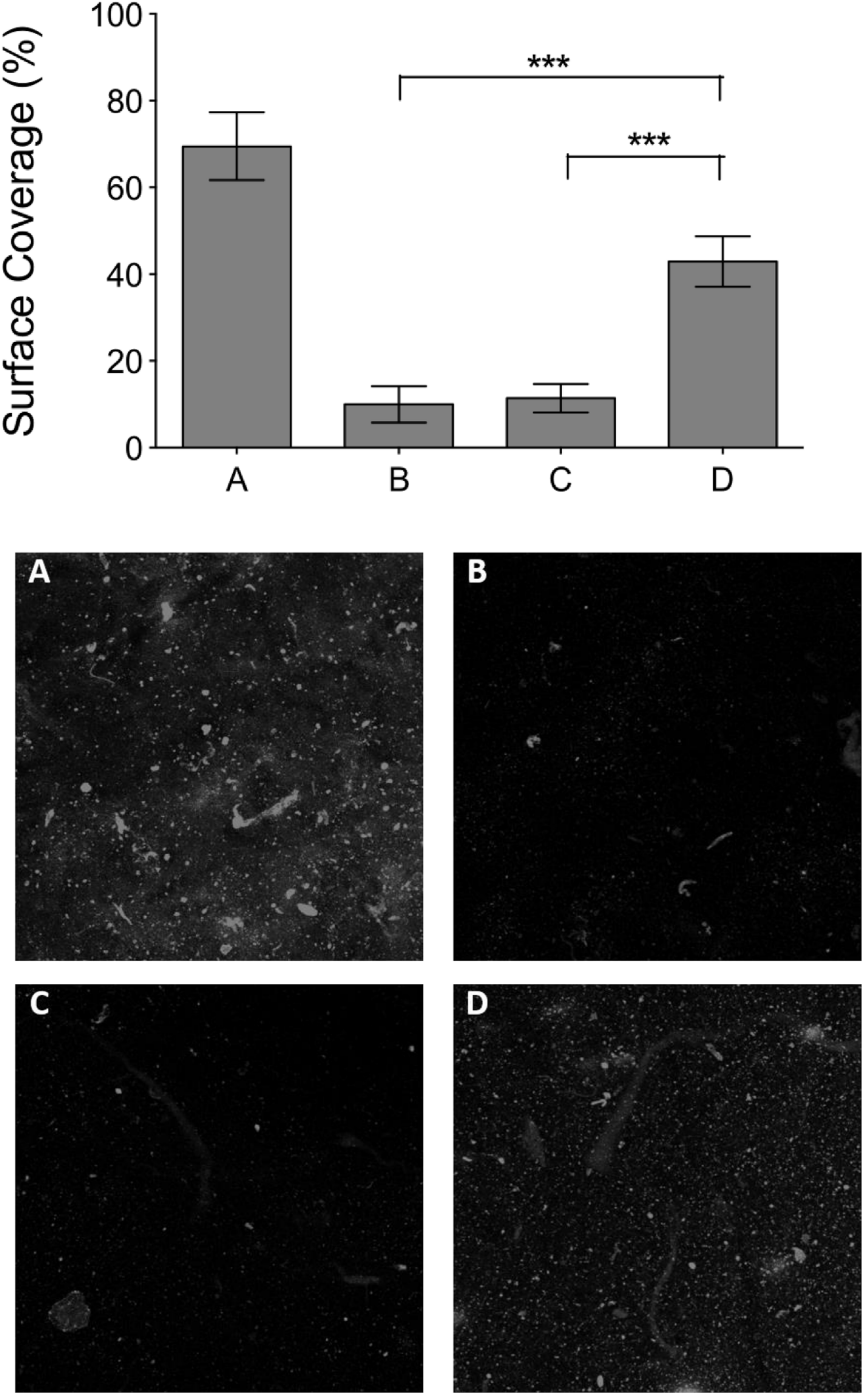
Platelet aggregation on derivatised EDC/NHS crosslinked collagen films. DiOC_6_-labelled human whole blood was perfused at a 1000 s^−1^ shear rate on non-crosslinked collagen films (A), 100% crosslinked films (B), 100% crosslinked films incubated with Diaz-ES-VWFIII_Nle_ in the dark (C) or 100% crosslinked films incubated with Diaz-ES-VWFIII_Nle_ after UV treatment for 5 min (D). Z-slices from the collagen film to the height of the thrombus were acquired on a confocal microscope. The thrombus mean surface coverage is calculated as a percentage of surface covered by fluorescent platelets. This graph shows pooled results from independent experiments using 4 different donors (p < 0.0001, 1-way ANOVA). Diaz-ES-VWFIII_Nle_ followed by UV treatment led to a significant increase in platelet aggregation on 100% crosslinked films. Representative z-stacks showing thrombus formation on collagen films for each conditions are shown below.

Human blood, anticoagulated and labelled as described in section 2.9, was perfused at a shear rate of 1000 s^−1^ over collagen films, and Z-stacks of images of the platelet thrombi were acquired. “Flattened” Z-stack projections were generated for 6 fields of view for each condition in four independent repeats, and representative images are shown in Fig. 7. Surface coatings used were non-crosslinked collagen films (Fig. 7 A), or films crosslinked to 100%, then either with no further treatment (Fig. 7 B), or treatment with Diaz-ES-VWFIII_Nle_ without (Fig. 7 C) or with (Fig. 7 D) UV exposure. Substantial platelet deposition can be seen only on non-crosslinked collagen films and crosslinked films derivatised with Diaz-ES-VWFIII_Nle_ after UV treatment. In contrast, crosslinked films without peptide (Fig. 7 B) or with Diaz-ES-VWFIII_Nle_ without UV (Fig. 7 C) did not support measurable platelet adhesion or thrombus formation.

On each Z-stack projection, the percentage of film surface covered by platelets was measured. Platelets readily formed thrombi on non-crosslinked collagen films, covering 69.5 ± 19.1% of the film surface. Platelets did not adhere to 100% crosslinked films, with only 5.6 ± 6.2% surface coverage. Treatment with Diaz-ES-VWFIII_Nle_ without UV exposure was not able to significantly restore thrombus formation (11.4 ± 9.4% surface coverage). In contrast, Diaz-ES-VWFIII_Nle_ with subsequent UV treatment supported substantial restoration of platelet surface coverage (42.9 ± 16.4%). However, thrombus size remained qualitatively smaller in volume and in height compared to non-crosslinked films. Nevertheless, surface coverage measurement on four independent repeats were pooled and showed significant increase in thrombus formation with Diaz-ES-VWFIII_Nle_ followed by UV exposure to 100% crosslinked collagen film untreated or treated with Diaz-ES-VWFIII_Nle_ without UV exposure (Fig. 7, p < 0.001).

### 3.9 HUVEC LDL uptake on derivatised 100% crosslinked films

**Fig. 8:**
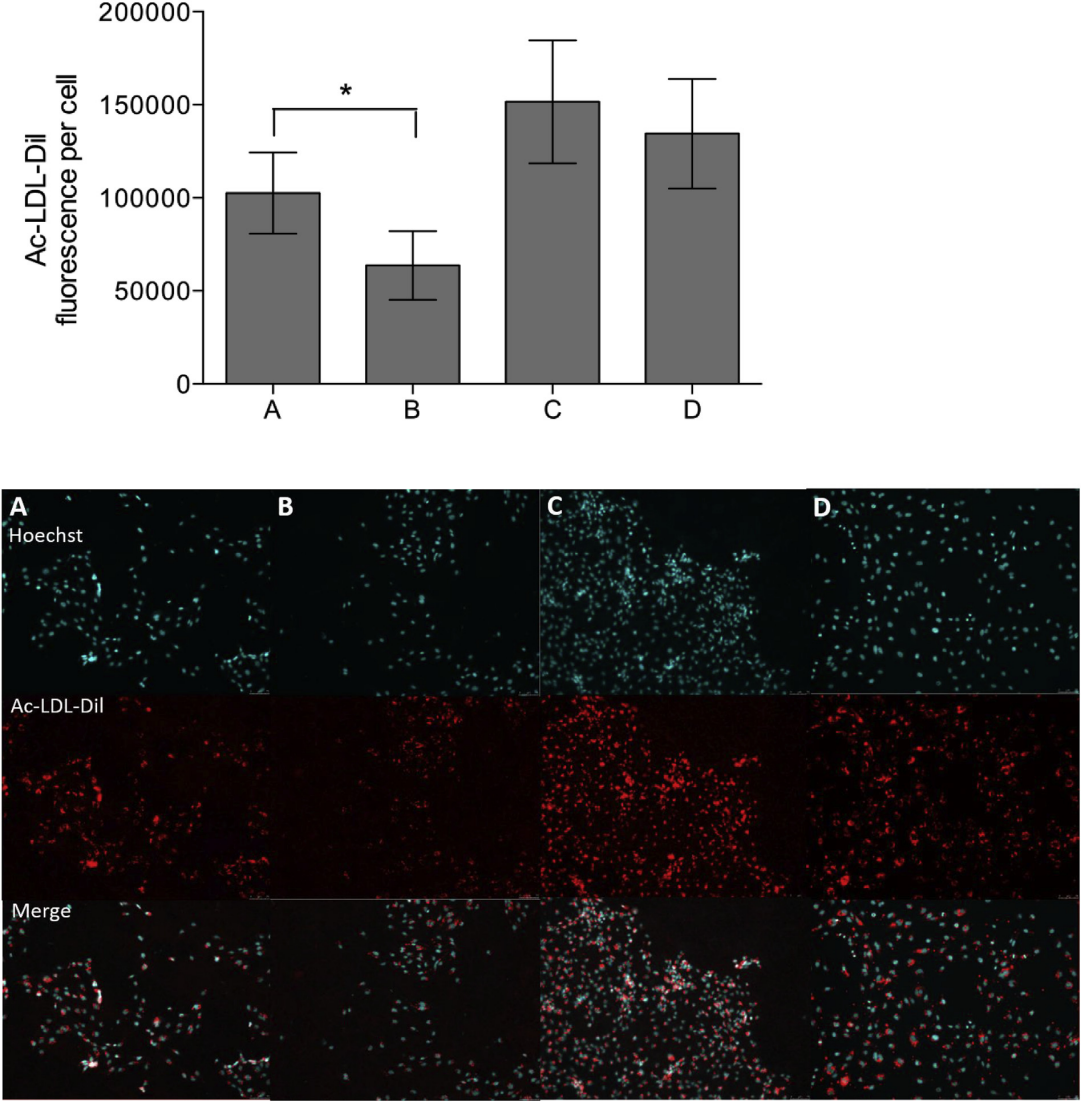
Ac-LDL uptake on HUVECs on derivatised EDC/NHS crosslinked collagen films. HUVECs were cultured in Ac-LDL-Dil-containing EGM-2 cell culture media on 100% crosslinked films incubated with Diaz-ES-VWFIII_Nle_ after UV treatment for 5 min (A), 100% crosslinked films (B), non-crosslinked films (C) and plastic culture dish (D). Cell nuclei were stained with Hoescht 33342 after fixing. The graph above shows the mean raw intensity value per cell obtained from 10 randomly selected fields of view in 8 independent repeat (p < 0.01, 1-way ANOVA). Diaz-ES-VWFIII_Nle_ followed by UV treatment led to a significant increase in LDL uptake in HUVECs. Representative fields of view for each condition are shown below.

HUVECs were incubated on derivatised collagen films with culture media containing Dil-labelled Ac-LDL. After 24 h, LDL uptake was observed in HUVECs incubated on 100% crosslinked films with UV-linked Diaz-ES-VWFIII_Nle_ (Fig. 8A), untreated 100% crosslinked films (Fig. 8B) and on non-crosslinked films (Fig. 8C), with accumulation Ac-LDL-Dil around the cell nuclei stained with Hoechst 33342. An additional control with HUVECs left on tissue culture plastic was also added (Fig. 8D). The amount of Ac-LDL-Dil internalized per cell was measured by fluorescence on 10 randomly selected fields of view per conditions, in each of 8 independent repeats. The presence of covalently linked Diaz-ES-VWFIII_Nle_ significantly increased LDL uptake in HUVECs (102597 ± 21876 arbitrary units of fluorescence intensity, one-way ANOVA, p < 0.01) compared to untreated 100% crosslinked films (63683 ± 18455), while remaining lower than on non-crosslinked films (151580 ± 32967) or empty culture dish (134518 ± 29455). This experiment indicates higher endothelial cell activity on crosslinked collagen films derivatised with covalently linked THPs (one way ANOVA, p < 0.01).

## 4 Discussion

EDC/NHS crosslinking creates covalent bonds between lysine residues and glutamate or aspartate residues within the collagen sequence [47]. This affects integrin-binding sites directly, which are composed of a Gxx'GEx'' generic sequence [13], and the resulting loss of integrin binding was demonstrated in our previous work [[8], [9], [10],^15^]. The binding site for DDR2 and VWF A3 was located within residues 572 to 583 of the collagen helix using homotrimeric collagen II and III Toolkits. It contains the sequence GARGQOGVMGFO in collagen II and GPRGQOGVMGFO in collagen III. Neither contains any residues likely to form crosslinks after EDC/NHS treatment. Collagen I, however, used to form the films in this study, is a heterotrimer, and the corresponding site, GARGQ**A**GVMGFO, in its α1 chain is not competent to bind VWF A3, since it lacks a critical hydroxyproline residue. A composite VWF-binding site has been proposed [48] which requires all three strands of collagen I, and the α2 chain contributes the missing O residue. Crucially, the corresponding α2 chain sequence, GARGEOGNIGFO, contains a glutamate residue that, if crosslinked, would obstruct incoming binding partners including DDR2 and VWF A3. This binding site in collagens I, II and III is also adjacent to a reactive lysine (K586 in the collagen helix). Chemical crosslinking of this nearby residue might also restrict access of the collagen-binding proteins, VWF and DDR2.

First, we demonstrated that increasing EDC/NHS treatment of collagen films affects their affinity for DDR2 and VWF, which gradually decreases and reaches a minimum at the standard crosslinking conditions (defined as 100%). There is therefore a need to restore DDR2 and VWF A3 binding by incorporating photo-activatable triple-helical peptide ligands that can be irreversibly covalently linked to collagen.

We adapted the method of Khew et al. [37] to synthesise THP ligands. Peptide strands were elongated on resin, with an Ahx linker added at the N-terminus to provide flexibility to favour subsequent end-stapling. This linker will also further separate the end-stapling site and the active binding site of the THP, thus limiting undesired interactions between the two. End-stapling was carried out by coupling the tri-acid hexapeptide Fmoc-GFGEEG to three resin-supported strands. Addition of N-terminal templates in an attempt to covalently link the three peptide strands is known to be a difficult and slow reaction [49]. Previous attempts in our lab to end-staple peptides at their C-terminus by introducing two lysine residues gave poor results. The end-stapling strategy presented here has two main advantages. Firstly, it stabilises the active triple-helical conformation increasing the transition temperature by 4 °C (or 9 °C for an end-stapled THP containing GFOGER) [15]. Secondly, it provides a single reactive site, the primary amine located at the end-terminus side, per end-stapled THP. Molecules of interest, such as fluorophores, or, in this case, diazirine, can then be grafted to THPs with a 1:1 stoichiometry. Diazirine is a photoactive group able to react readily with any carbon skeleton after UV exposure, making this technology applicable to a wide range of substrates. Furthermore, in contrast to usual strategies for grafting ligands, this allows collagen film derivatisation with pre-assembled and functional THPs.

We used the conditions for covalent linkage of photoreactive THPs that were previously optimised for the integrin-binding THP, GFOGER [15], where, after 5 min of UV treatment and at a THP concentration of 5 μg/ml, maximal and specific covalent linkage of the photoreactive THP was observed. We anticipate the same specific reaction here, using GPRGQOGVNleGFO. DDR2 and VWF binding to films derivatised in this way was assessed using recombinant DDR2-Fc and VWF A3-GST fusion proteins. In both cases, best binding was obtained after treatment with diazirine-containing peptides, and only after UV exposure. Compared to crosslinked collagen not treated with peptides, the binding of VWF A3 and DDR2 increased seven-fold and three-fold respectively. Increased affinity was also observed without UV treatment or in the presence of non-photoreactive peptides. This may be due to passively-adsorbed peptides still present despite stringent washings. Nonetheless, for long-term applications, covalent attachment to our collagen substrate remains desirable to eliminate peptide elution.

We examined the function of immobilised THPs by seeding cells transfected with DDR2-Flag on derivatised crosslinked collagen films, then detecting the phosphorylation of tyrosine 740 (Y740) on the intracellular domain of DDR2 [50]. This regulatory residue is a target for the intracellular tyrosine kinase c-Src, and when phosphorylated, Y740 permits the autophosphorylation and activation of DDR2. It is widely used to report DDR2 activation. Immunofluorescence and Western blot experiments allowed us to investigate both DDR2 binding to derivatised films and its activation state.

On all collagen films tested (regardless of crosslinking status, peptide and UV treatment), transfected COS-7 cells were found to express similar levels of DDR2-Flag, as expected. Film functionalization with photo-activated THP resulted in significant Y740 phosphorylation after UV exposure, reaching levels similar to those observed on non-crosslinked films. Western blots of HEK293 cell lysates confirmed that anti-Flag and anti-pY740 antibodies specifically bound to DDR2, which appeared as one, two or three bands between 125 and 130 kDa. Meanwhile, although high levels of DDR2 expression were detected on cells incubated on crosslinked collagen films, very little Y740 phosphorylation could be seen. Occasional tyrosine phosphorylation was also observed on films treated with VWFIII_Nle_ or Diaz-ES-VWFIII_Nle_ without UV, likely due to residual passively-adsorbed peptides. This observation is consistent with our DDR2-Fc binding assay on derivatised films (Fig. 3 B), where passive peptide absorption accounted for high DDR2-Fc binding.

Nevertheless, quantification of DDR2-Flag and phosphorylated DDR2, using both Western blot and immunofluorescence techniques, showed that Diaz-ES-VWFIII_Nle_ and UV treatment were both required to restore DDR2 activation on crosslinked films to a level comparable to that seen on native collagen. This result confirms that covalent linkage of DDR2-binding peptides on EDC/NHS crosslinked films can restore receptor binding and promote receptor activation.

As we have shown in previous studies, EDC/NHS treatment resulted in a drastic loss of integrin mediated cell adhesion and spreading, which can be restored by grafting Diaz-ES-GFOGER. However, the immunofluorescence experiment presented here was designed to evaluate DDR2 function only and did not investigate integrin activity. Therefore, differences in cell morphology were observed between non-crosslinked films (where cells are spread on the film surface) and 100% crosslinked films derivatised with Diaz-ES-VWFIII_Nle_ (where cells remain round). Part of our on-going work is to graft both photoreactive GFOGER and GPRGQOGVNleGFO containing THPs on films, to study the joint effect of peptide targeting integrins and DDR2. This is of particular interest since DDR receptors are known to up-regulate integrin affinity [20]. Together, such cooperative processes may increase further the effects of covalently linked peptides.

The experiments described above were conducted using model systems that are easily controlled but lack an immediate application. To develop the field beyond this proof of principle, we applied GPRGQOGVNleGFO-derivatized films to the study of human platelets and endothelial cells, each of which might represent important targets in biomaterial design. First, we studied the binding of VWF, as the recombinant A3 domain and as the full length protein in the setting of whole blood, and its effect on platelet aggregation. In vascular injury, thrombus formation is initiated by the association of platelets with exposed subendothelial collagen via VWF recruited from the blood plasma [30]. VWF is a key component of haemostasis and subsequent blood vessel repair and could play an important role in regenerative medicine or acute surgical applications. Compared to the binding of the A3 domain of VWF, full-length VWF static adhesion assays gave very low signals (data not shown). This was anticipated, since full-length VWF needs sufficiently high shear in order to form multimers and bind efficiently to its ligand [51]. The binding of platelets under flow on derivatised collagen films in the presence of full-length VWF was therefore investigated. Several protein–protein interactions are known to be involved in thrombus formation, including VWF binding to GPRGQOGVMGFO and homologous sequences in the collagens, immobilised VWF binding to platelet GpIb, integrin α2β1 binding to GxOGER motifs and GPVI binding to GPO repeats [21]. To approximate physiological conditions, a shear rate of 1000 s^−1^ was used for perfusion of whole blood over collagen films, which corresponds to arterial flow conditions. Platelets were selectively marked with DiOC_6_, a fluorescent dye, allowing us to follow their deposition on collagen films by fluorescence. Extensive platelet aggregation could be detected on non-crosslinked collagen films. In contrast, EDC/NHS crosslinking ablated both VWF and integrin binding, as discussed earlier, resulting in a total loss of platelet adhesion. We assume GPO repeats not to be affected directly by EDC/NHS crosslinking, as recombinant GPVI showed no loss of binding on collagen films crosslinked to various degrees (data not shown).

Films treated with Diaz-ES-VWFIII_Nle_ but not exposed to UV light supported only slight platelet deposition, suggesting that passively-bound peptides were removed under shear. However, treatment with Diaz-ES-VWFIII_Nle_ and UV restored platelet surface coverage to about 50% of the level on native collagen films, although under these conditions thrombi were smaller. Integrin α_2_β_1_-dependent binding will remain ablated by EDC/NHS treatment, with the loss of Gxx'GEx'' motifs. Our findings are consistent with a synergic effect between GPVI, VWF and integrins being required for full thrombus formation [21]. In our current study, only GPVI (through GPO repeats in the collagen) and VWF (through GPRGQOGVNleGFO) binding occurs, but not integrin binding, leading to only partial thrombus formation. We observed a similar effect previously, where platelets were perfused on slides coated with GFOGER-, GPO- or GPRGQOGVNleGFO-containing peptides [21]. We expect that full restoration of platelet aggregation would require derivatisation of crosslinked films with both GPRGQOGVNleGFO- and GFOGER- containing THPs, an experiment for the future. Such a material might be valuable in stents used to treat aortic aneurysm, for example, where promotion of blood clotting stabilises the distension of the aorta. Use of an insoluble biomaterial would be a means of constraining clotting to the desired location.

The last step of this study was to evaluate the feasibility of transposing THP-derivatised collagen substrates to tissue engineering applications. Endothelial cells have the ability to undergo angiogenesis, the process of formatting new blood vessels from pre-existing vasculature, which is essential for tissue repair at the site of injury. These cells are therefore prominent candidates for seeding on biomaterials. As a consequence, it was important to ensure that endothelial cells could fulfil their physiological function on our derivatised crosslinked collagen films. We selected HUVECs for this purpose for their convenience and because they are widely used in experimental settings. The uptake and regulation of Ac-LDL is a widely used marker to assess the activity of endothelial cells. We demonstrated that covalently linked Diaz-ES-VWFIII_Nle_ THPs were able to stimulate Ac-LDL internalization in HUVECs on 100% crosslinked films. The patterns observed in the presence of peptides is similar to what is observed on empty tissue culture treated plastic dishes, with Ac-LDL accumulation in the cytoplasm, indicating normal functioning of the cells. Again, some notable difference could be observed between results on derivatised crosslinked films and non-crosslinked films. Diaz-ES-VWFIII_Nle_ restores LDL uptake only partially, and additional ligands for other collagen binding receptors might be required for full HUVEC activity. At the moment, we can only speculate on how the THP ligands can beneficially stimulate HUVECs and we are currently investigating the repertoire of collagen receptors expressed on their surface to provide insight on these mechanisms. Nevertheless, our data highlights the potential of derivatised films for use as a biomaterial promoting angiogenesis and tissue regeneration.

The present work, together with our previous study, offers a means of conferring collagen-binding integrin-, VWF- and DDR-reactivity upon an inert substrate. GPRGQOGVNleGFO is known to bind not only VWF and DDR2, but also DDR1 and SPARC (Secreted Protein Acidic and Rich in Cysteine) [20,52]. We therefore expect this peptide to be able to promote DDR1 and SPARC recruitment to crosslinked films, increasing the restoration of physiological function to cross-linked collagens in tissue engineering. The scope of the method developed here can also be extended to include three-dimensional matrices [10] and to other inert substrates, not necessarily collagen-based [53], due to the broad reactivity of the diazirine group. Other collagen motifs for other receptors, for example for GPVI or OSCAR, are currently being studied in order to broaden the methodology further to allow enhancement of reactivity beyond that of native collagen. A wider range of THP ligands will allow us to adjust cell activity for different cell types to particular applications in tissue engineering.

## 5 Conclusion

Our objective is to promote cell activity on biomaterials, especially for use in regenerative medicine, by grafting THPs containing collagen-binding sites onto inert substrates. We have previously reported a methodology to derivatize EDC/NHS crosslinked films with photoreactive THPs. This resulted in increased cell reactivity towards the collagen substrate, illustrated by cell binding and spreading, after attaching a GFOGER-containing THP that supports the attachment of collagen-binding integrins. Here, we diversified our range of photoreactive ligands to address different collagen-binding receptors for a wider range of applications. Of particular interest is Diaz-ES-VWFIII_Nle_, containing the GPRGQOGVNleGFO active sequence, which targets both DDR1 and DDR2, and VWF. DDRs have been implicated in wound healing and cell migration, and DDR2 has been reported in epicardium-derived cardiac fibroblasts [29] and cardiac mesenchymal stem cells [28]. The proper regulation of these receptors is likely to be important to direct cell behavior and regeneration in several tissue engineering settings. VWF can equally present multiple interests in vascular repair, as it is a key factor in platelet thrombus formation in flowing blood. We have demonstrated that Diaz-ES-VWFIII_Nle_-derivatised films had an increased affinity for recombinant DDR2 and VWF A3. Beyond simple binding activity, we have shown that receptors could be activated by our THPs covalently bound to collagen films, which was highlighted by tyrosine phosphorylation of DDR2 in transfected cells, and platelet deposition in human blood. Finally, we have demonstrated that derivatised collagen films could support HUVECs activity, a key step towards the production of collagen-based structures promoting angiogenesis. In this way, we have overcome the loss of reactivity that occurs when collagen is crosslinked to enhance its mechanical properties, paving the way for the construction of collagen scaffolds and other biomaterials for diverse applications.

## Data availability

The raw/processed data required to reproduce these findings cannot be shared at this time due to technical or time limitations.
