# Supplementary material for: Coupling of a specific photoreactive triple-helical peptide to crosslinked collagen films restores binding and activation of DDR2 and VWF
Source: Biomaterials. 2018 Nov;182:21–34. doi: 10.1016/j.biomaterials.2018.07.050 (PMC6131271; doi:10.1016/j.biomaterials.2018.07.050)

**Supporting Info**

S1. Mass spectra

MALDI spectrum of VWFIII_Nle_


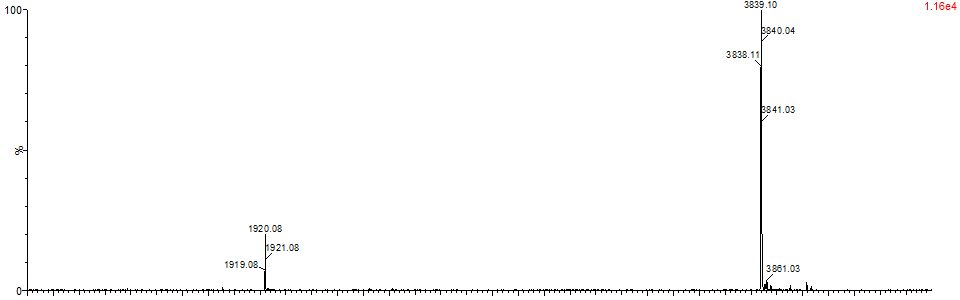


MALDI spectra of Diaz-ES-VWFIII_Nle_

Full spectrum


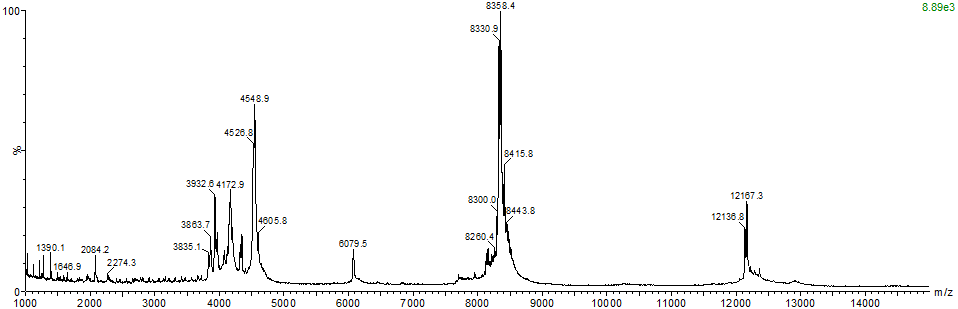


Close-up on the 11500-13500 m/z region

S2. Transition temperature measurement by polarimetry


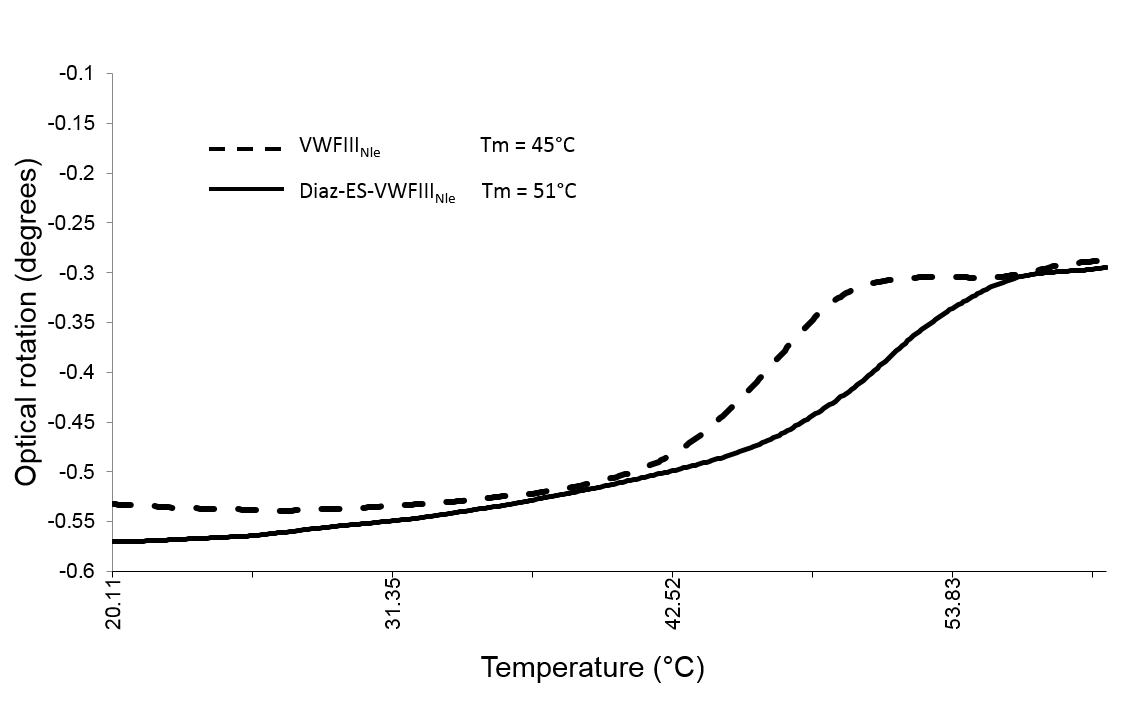

Supplement: Multimedia component 2 [file mmc2.docx]
